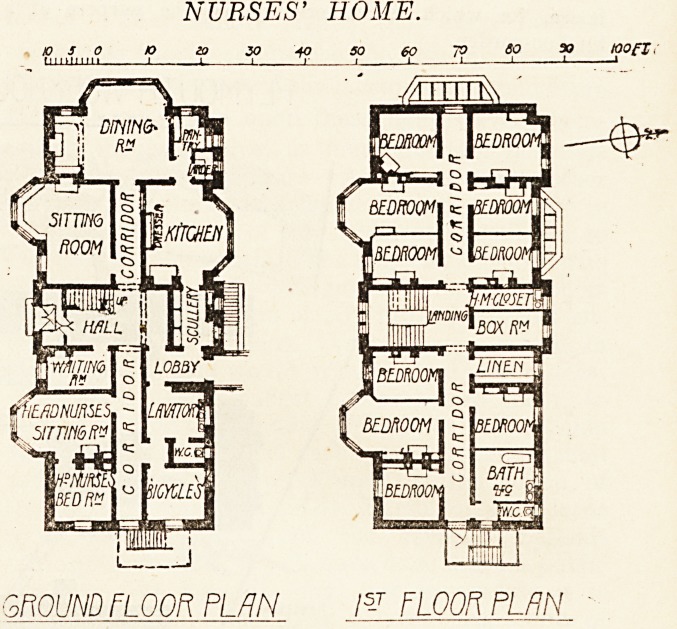# Plymouth Workhouse New Infirmary

**Published:** 1910-01-22

**Authors:** 


### PLYMOUTH WORKHOUSE NEW INFIRMARY.

THE new Infirmary has been erected on a site of about three acres in extent immediately adjoining the workhouse, for which it formerly served the purpose of a kitchen-garden.

PLYMOUTH WORKHOUSE · NEW INFIRMARY·

BLOCK PLAN·

The Infirmary consists of six detached blocks connected together and with the workhouse by glazed covered ways. These blocks are: (1) Administration, (2) Nurses' Home, (3 and 4) Male and Female General Ward Blocks, (5) Venereal Block, and (6) Maternity Block.

PLYMOUTH WORKHOUSE · NEW INFIRMARY·

GROUND FLOOR PLAN OF MALE WARDS (1ST FLOOR SIMILAR)

BLOCK A·

GROUND FLOOR PLAN OF FEMALE WARDS (1ST FLOOR SIMILAR)

BLOCK B·

The Administration Block is a compactly-planned one-story building, containing the receiving-rooms for male and female patients, the office, sitting-room and bedroom for the Superintendent Nurse, Medical Officer's room, the operation room, anæsthetic room, and the dispensary.

The Nurses' Home is a three-story building, having separate bedrooms for twenty nurses and one head nurse.

On the ground floor are the dining-room with kitchen offices adjoining a large sitting-room, waiting-room, lavatory, and bicycle-room. The provision of two bath-rooms for twenty-one nurses is hardly sufficient. An average of one bath-room to eight nurses is not at all an excessive proportion. With this exception the building seems admirably planned for its purpose.

The general ward blocks are of the usual Local Government Board type with coupled beds and the meagre airspace in the wards which was thought sufficient half a century ago, and which has never been increased, notwithstanding the fact that the class of case treated in these infirmaries has tended to become more and more severe. In this particular plan the position of the sanitary blocks, which is evidently determined by the intention to make them available for the separation wards as well as the general wards, interferes unfortunately with the cross ventilation at the end of the ward. The cut-offs to the w.c.'s are not in all cases what they should be. The whole arrangement in point of fact is crippled and starved by the obsolete regulations of the Local Government Board.

The Maternity Block is one story in height and contains a ward for ten beds, two labour wards with a ward-kitchen and the usual sanitary offices.

To supply both workhouse and infirmary a new boiler-house has been erected with two large steam boilers and an economiser. Steam pipes are carried to the various blocks to calorifiers fixed in the basements whence the

*NURSES' HOME.*

hot water is distributed to the various fittings and radiators.

The whole of the work was designed by and carried out under the superintendence of Messrs. Thornely and Rooke, architects, of Plymouth.

## Figures and Tables

**Figure f1:**
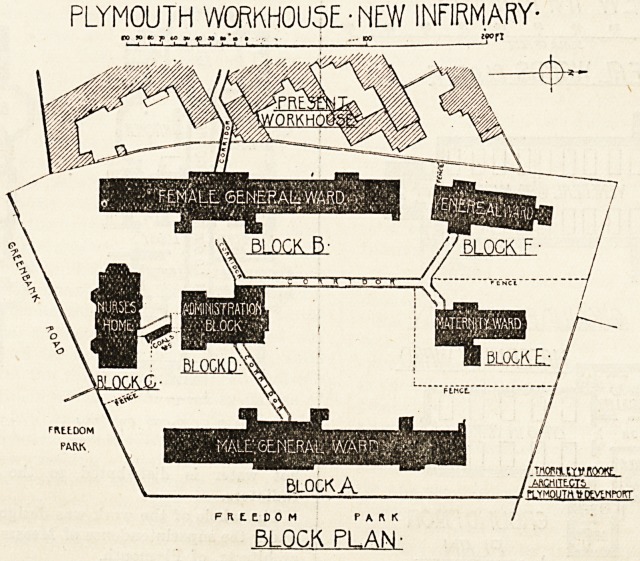


**Figure f2:**
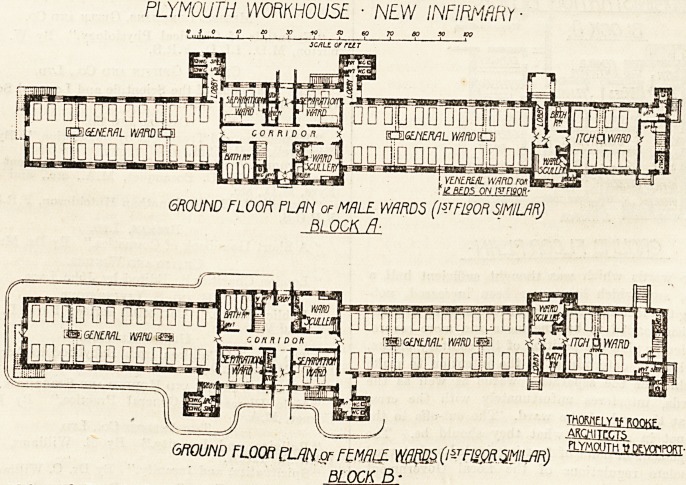


**Figure f3:**
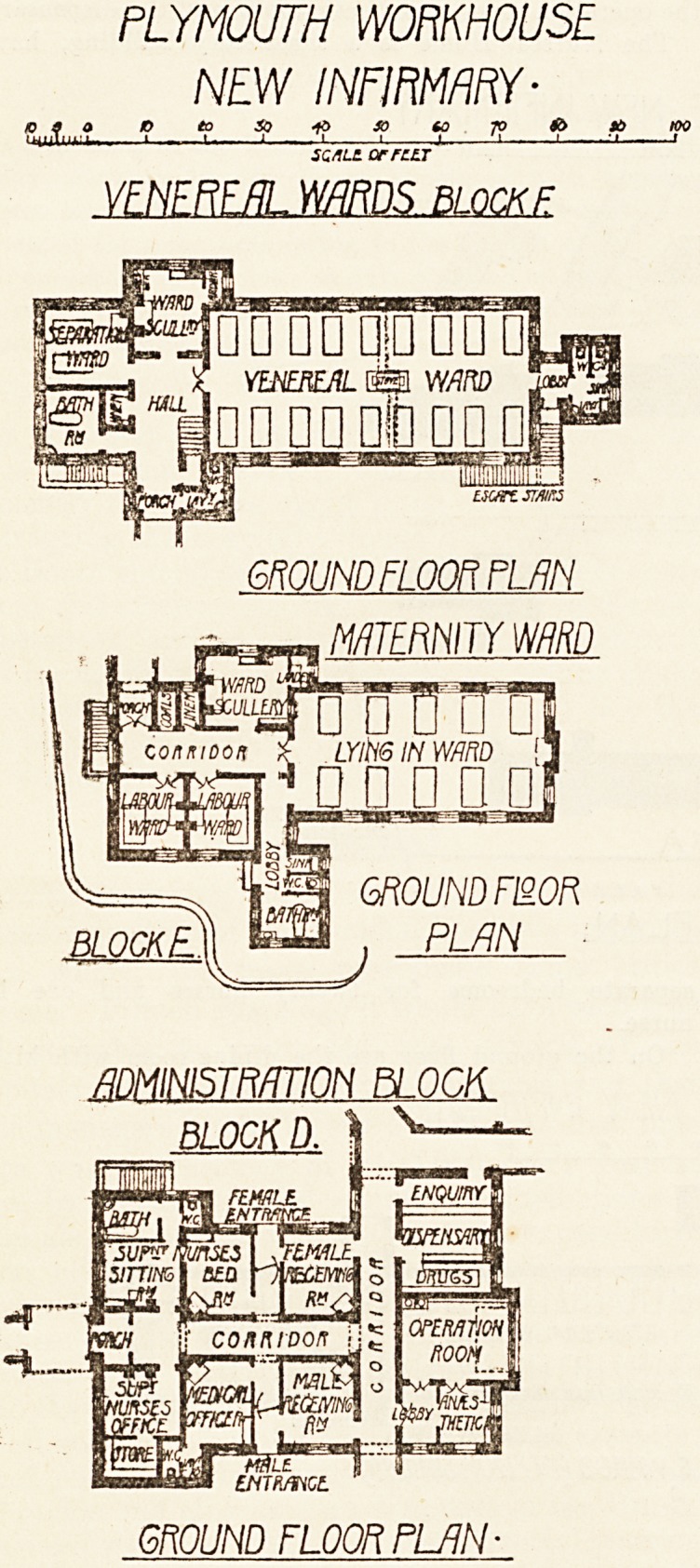


**Figure f4:**